# Synthesis and Characterization of Ultra‐Small Gold Nanoparticles in the Ionic Liquid 1‐Ethyl‐3‐methylimidazolium Dicyanamide, [Emim][DCA]

**DOI:** 10.1002/open.202300106

**Published:** 2023-08-31

**Authors:** Jana Hildebrandt, Andreas Taubert, Andreas F. Thünemann

**Affiliations:** ^1^ Bundesanstalt für Materialforschung und -prüfung (BAM) Unter den Eichen 87 12205 Berlin Germany; ^2^ Institute of Chemistry University of Potsdam 14476 Potsdam Germany

**Keywords:** gold cluster, ionic liquid, small-angle scattering, reference material

## Abstract

We report on gold clusters with around 62 gold atoms and a diameter of 1.15±0.10 nm. Dispersions of the clusters are long‐term stable for two years at ambient conditions. The synthesis was performed by mixing tetrachloroauric acid (HAuCl_4_ ⋅ 3 H_2_O) with the ionic liquid 1‐ethyl‐3‐methylimidazolium dicyanamide ([Emim][DCA]) at temperatures of 20 to 80 °C. Characterization was performed with small‐angle X‐ray scattering (SAXS), UV‐Vis spectroscopy, and MALDI‐TOF mass spectrometry. A three‐stage model is proposed for the formation of the clusters, in which cluster growth from gold nuclei takes place according to the Lifshitz‐Slyozov‐Wagner (LSW) model followed by oriented attachment to form colloidal stable clusters.

## Graphical abstract

Formation of 1 nm‐sized gold clusters with long‐stability in an ionic liquid is reported. Formation via a three‐stage process is suggested. Cluster growth from gold nuclei takes place according to the Lifshitz–Slyozov–Wagner (LSW) model followed by oriented attachment to form colloidal stable clusters.

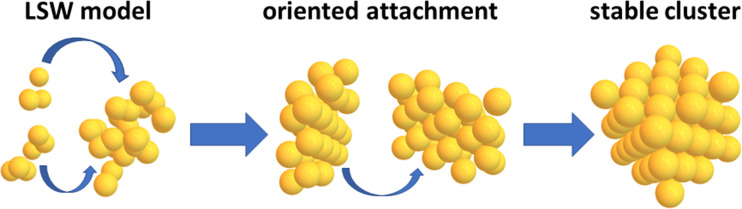

## Introduction

Gold clusters are of great current interest due to their unique structural features,^[1]^ their electronic and optical properties,[^2^, ^3^] application potential in catalysis[^4^, ^5^] and medicine.[^6^, ^7^] Metal clusters are generally defined as small (≤1 nm) particles that contain 2–1000 metal atoms, as opposed to the considerably bigger nanoparticles, being 1–100 nm in diameter. A key aspect in cluster synthesis is that after the reduction of the precursor, usually Au(III), to the Au(0) species further growth of the nuclei (towards the much larger nanoparticles) must be suppressed.^[8]^ Careful choice of the reducing agent and the reaction conditions enable a highly controlled nucleation process,^[9]^ while the addition of stabilizers like thiolate, selenoate, phosphine, or acetylide ligands prevents further growth and aggregation.[^10^, ^11^] Depending on the ligand, different clusters are obtained: in the presence of thiolates Au_*n*_ clusters with n=15-940 form.^[12]^ Phosphines typically yield smaller clusters with n≤13 .^[12]^ Thus, the cluster size depends on numerous parameters and many (often contradicting) reports on the formation and stabilization of Au clusters exist.^[13]^ Surprisingly, the synthesis of metal clusters in ionic liquids (ILs) has not yet been investigated, although the synthesis of (much larger) metal nanoparticles in ILs is well documented.^[14]^ This is interesting because ILs are efficient reaction media and templates for the synthesis of a virtually unlimited number of inorganic compounds and materials. A typical cluster synthesis in conventional (molecular) solvents starts from Au(III) salts and uses various reducing agents to produce the Au(0) atoms, which likely then aggregate and form very small clusters. Although no true clusters have been made in ILs so far, a few reports describe the synthesis of very small Au nanoparticles. Redel et al.^[15]^ synthesized Au nanoparticles with diameters as low as 2 nm from a solution of KAuCl_4_ in the IL 1‐butyl‐3‐methylimidazolium tetrafluoroborate, [BMIm][BF4]. Jin et al. synthesized Au nanoparticles with a diameter between 2 and 10 nm in the thiol‐functionalized IL [1‐(2,3‐dimercapto‐acetoxypropyl)‐3‐methylimidazolium‐3‐mercapto‐1‐propanesulfonic acid] in the presence of H_2_O_2_ via a sonochemical approach^[16]^ and Hamm et al. produced Au nanoparticles via sputtering in 1‐ethyl‐3‐methylimidazolium ethyl sulfate, [EMIm][EtSO4], under argon flow.^[17]^ Wender et al.^[18]^ and Hatakeyama et al.^[19]^ employed plasma arc deposition and various sputtering approaches in ILs for metal nanoparticle synthesis. Finally, Mertens et al. synthesized very small *n*‐hexanethiolate‐protected gold particles with a diameter of ca 1.7 nm in various ILs.^[20]^ Despite the efforts to obtain and characterize clusters and nanoparticles in ILs, there are so far no reliable synthetic protocols enabling the comparison of calculations with experimental data. Consequently, there is essentially no knowledge on the formation and properties of Au clusters in ILs in spite of their high application potential in e. g. electronics or catalysis.

One of the most interesting, yet so far unresolved, questions in cluster chemistry in ILs is that some data indicate that the formation of clusters and nanoparticles in ILs may follow a completely different mechanism than in aqueous or organic media. Foremost, the ionic nature of ILs results in a preference for ionic solutes, and therefore ILs are often termed super dissociating solvents.[^21^, ^22^, ^23^, ^24^] Accordingly, small metal clusters synthesized in an IL may possess a different charge than those created in molecular solvents, and hence they may also have a completely different electronic structure. Since the electronic structure of metal clusters governs their physicochemical properties (such as their reactivity, magnetic, and optical properties),[^25^, ^26^] a change from molecular solvents to ILs may provide access to metal clusters with fundamentally different properties than clusters made via more conventional approaches. Moreover, the high charge at the ionic head groups of the IL components provides them with a coordination behavior that is different from molecular solvents. Finally, and again in contrast to molecular solvents, ILs have a particularly interesting nanostructure, which originates from the segregation of the polar and non‐polar moieties into separate domains.[^27^, ^28^, ^29^] The characteristic sizes of the resulting network of ionic and lipophilic domains depend, of course, on the particular IL. Consequently, it is highly likely that the stability, structure, and properties of the clusters are directly and very strongly influenced by this nonsegregated micro‐heterogeneous structure. Some of the structural features of the IL may even be imprinted into the clusters during synthesis.^[30]^ Combining all these aspects, it is clear that ILs are interesting tools to control and tune the structure and especially the physicochemical properties of metal clusters formed in these solvents. The focus of the current work is therefore to provide a first model system where clusters form in an IL and show long‐term stability. This model system should be easy and inexpensive to synthesize. The work is exploratory in regard that there is essentially no literature on cluster formation from ILs, and the data obtained from our study are intended to improve the understanding of the formation and tuning of very small inorganic entities in the sub‐nanometer to low nanometer size regime in ILs.

## Results and Discussion

**Figure 1:**
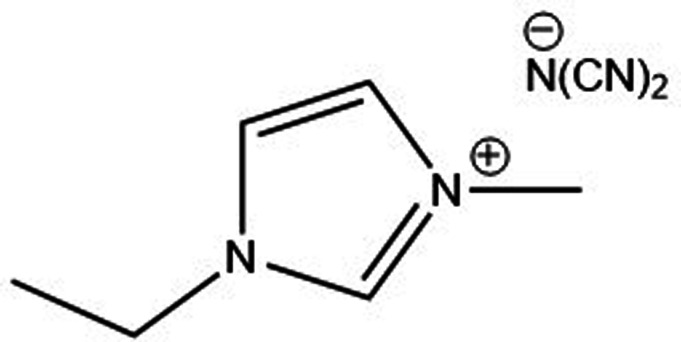
Chemical structure of the ionic liquid 1‐ethyl‐3‐methylimidazolium dicyanamide ([Emim][DCA]).

**Figure 2:**
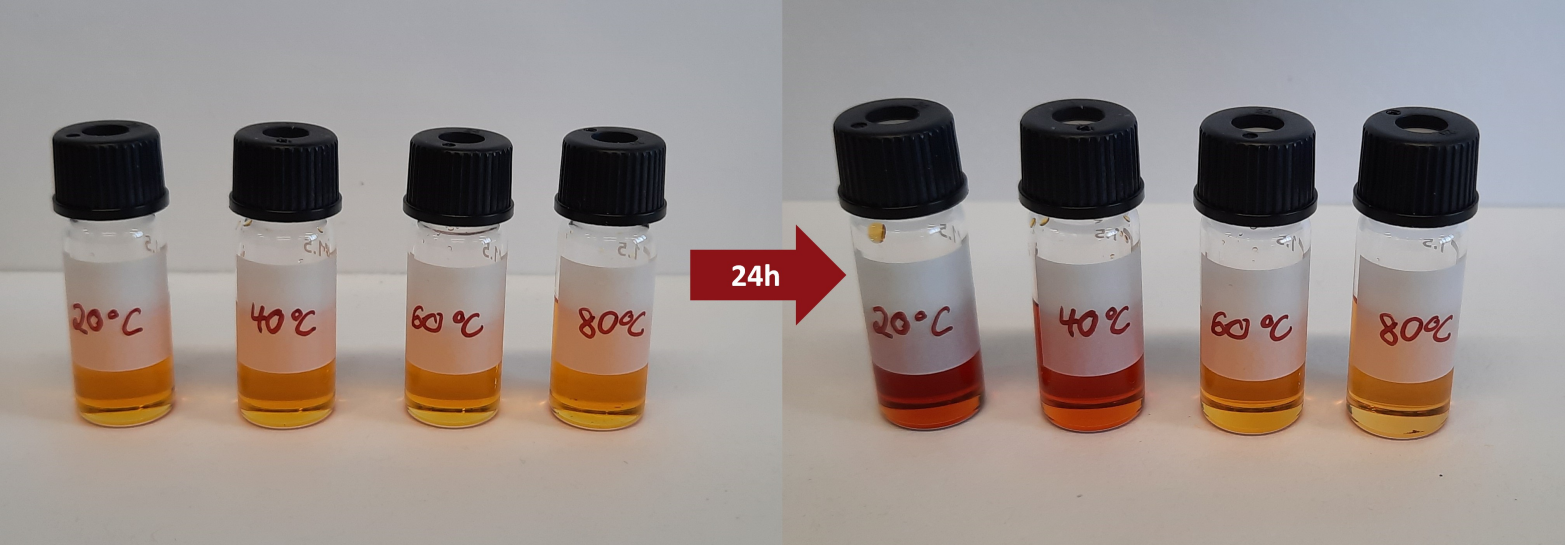
Vials with HAuCl_4_ dissolved in [Emim][DCA]. Photographs were taken directly after sample preparation (left) and after 24 h of reaction at 20, 40, 60 and 80 °C (right). The yellow to light orange solution turns red (20 °C), light red (40 °C), orange (60 °C) and light orange (80 °C) in dependence of the reaction temperature.

The salt HAuCl_4_ ⋅ 3 H_2_O was chosen as the first component for synthesis, as it has been proven to be highly suitable for the production of small gold nanoparticles.^[31]^ The second component selected was 1‐ethyl‐3‐methylimidazolium dicyanamide ([Emin][DCA]) as an IL with a low melting point of −21 °C and a low viscosity of η=21 cP at 25 °C.^[32]^ The chemical structure of [Emin][DCA] is displayed in Figure 1. The HAuCl_4_ ⋅ 3 H_2_O was dissolved in [Emin][DCA] for gold cluster synthesis and stirred for 24 hours at temperatures of 20, 40, 60, and 80 °C. The initial light yellow color of the mixtures changes with time depending on the temperature, ranging from light orange (80 °C) to red (20 °C), as shown in Figure 2. The color changes indicate that the gold salt reacts with [Emin][DCA], whereby the details of the reaction product seem to depend on the temperature. Next, we found no precipitate formed, and the solution remained homogeneous for two years. The absence of precipitation was surprising, as no surfactant was added for colloidal stabilization. Tentatively, we assume the following scenario for interpretation: The IL reduces the gold ions to gold atoms, which coalesce into clusters and subsequently are stabilized at low sizes by the IL. In the following, we attempt to prove this hypothesis by means of UV‐Vis and SAXS investigations.

### Optical properties

**Figure 3:**
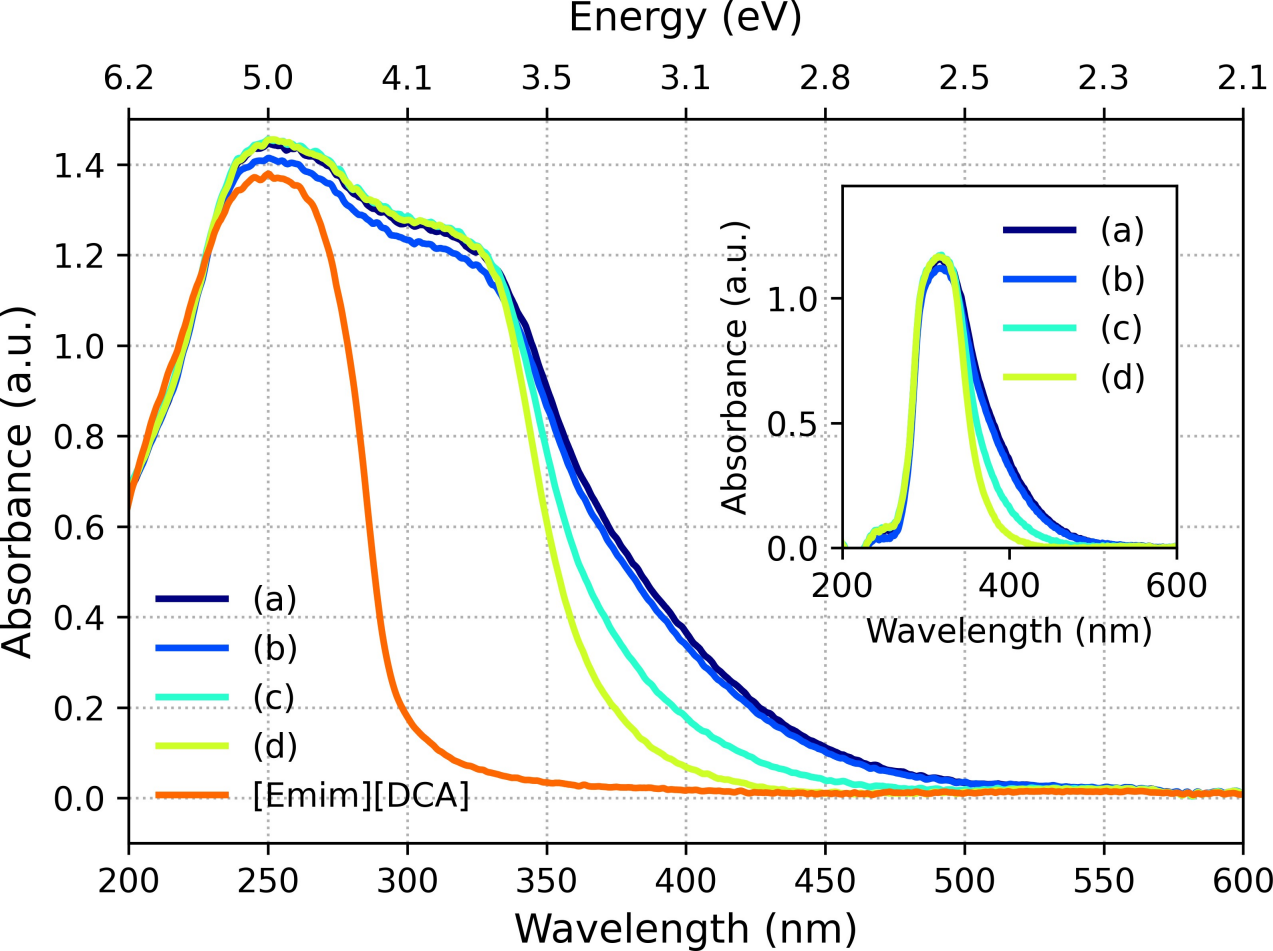
UV‐Vis absorption spectra of [Emim][DCA] and gold nanoclusters in [Emim][DCA] synthesized at temperatures of 20 °C (a), 40 °C (b), 60 °C (c) and 80 °C (d). Inset: Difference spectra, i. e. spectra in which the spectrum of [Emim][DCA] has been subtracted.

**Figure 4:**
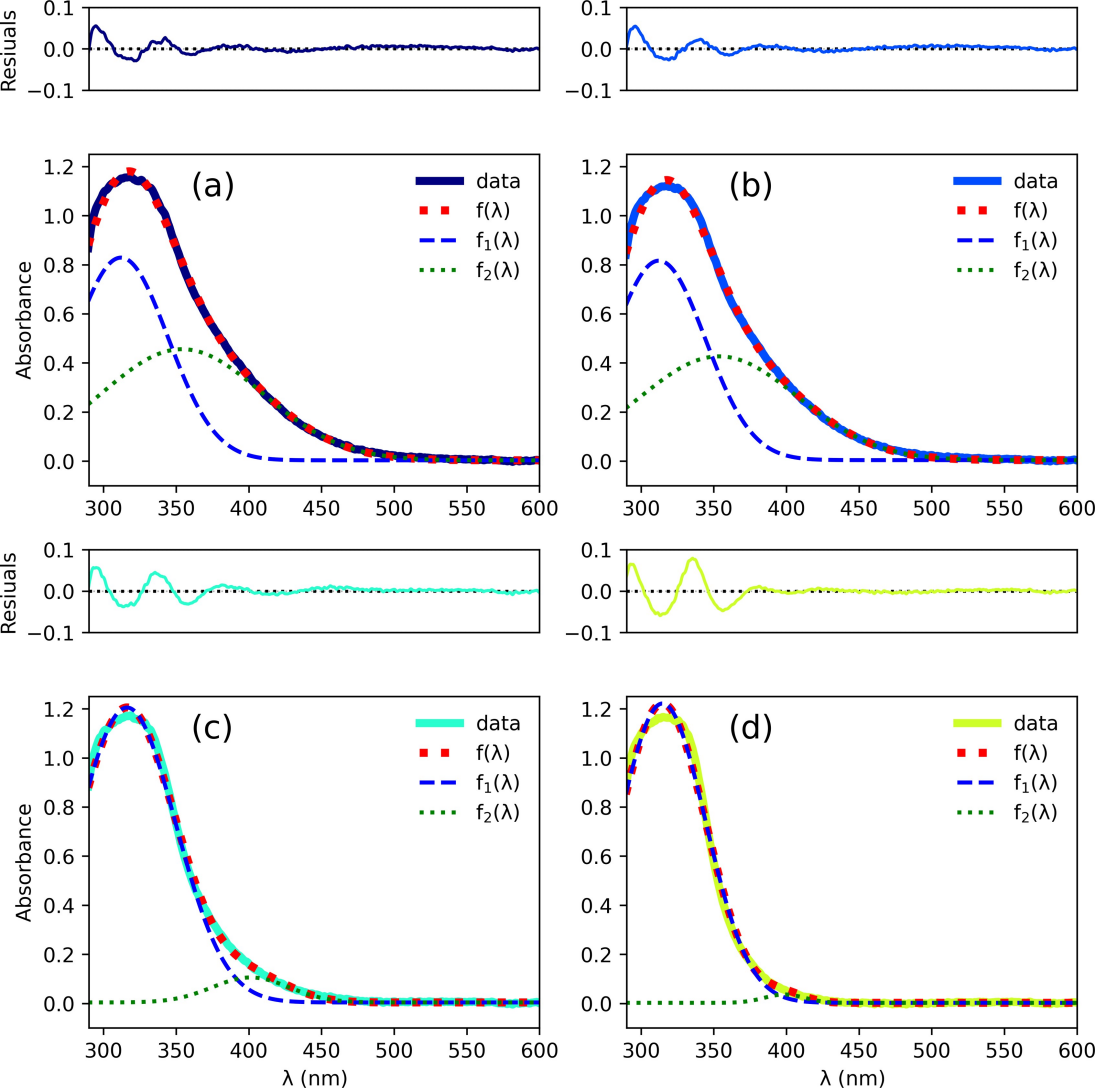
UV‐Vis absorption difference spectra (solid lines) and curve fits $f\left(\lambda \right)$ using Eq. (1) (red dotted lines) for clusters synthesized at 20 °C (a), 40 °C (b), 60 °C (c) and 80 °C (d). Contributions $f_{1}\left(\lambda \right)$ and $f_{2}\left(\lambda \right)$ are given (blue dashed and green dotted lines, respectively). The differences between the data and the model curves are shown above each panel.

**Figure 5:**
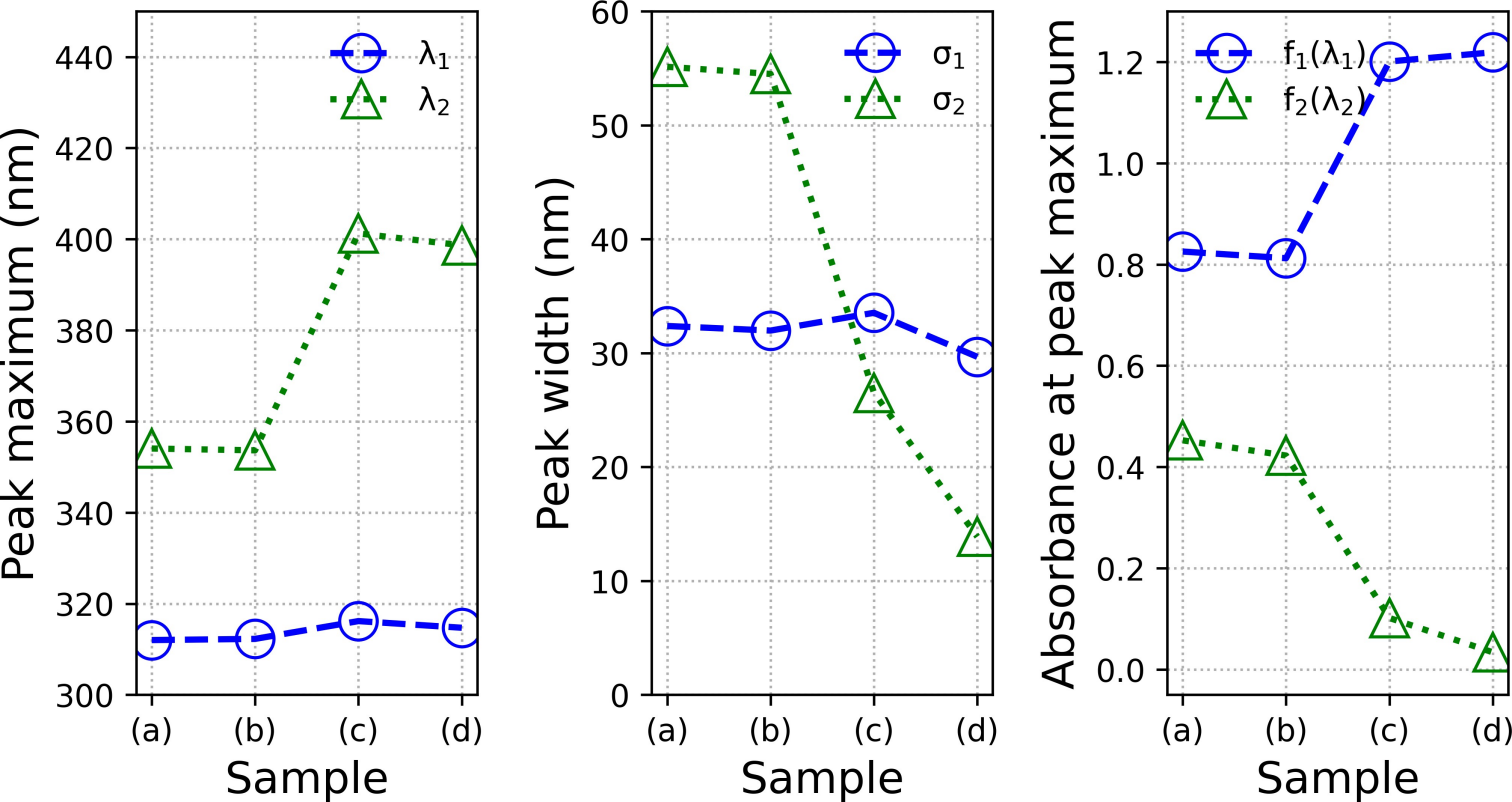
Parameters of curve fits of the UV‐Vis absorption spectra synthesized at 20 °C (a), 40 °C (b), 60 °C (c) and 80 °C (d).

The UV‐Vis absorption spectra of the samples are shown in Figure 3 (taken from the vials displayed in Figure 2). While the pure [Emin][DCA] has a strong absorption band at wavelengths below 300 nm with a maximum at 250 nm, the other samples additionally display a strong absorption at wavelengths larger than 300 nm. The maximum of 250 nm corresponds to an energy of 4.96 eV. This energy is in agreement with the HOMO–LUMO gap of [Emin][DCA] of (4.77±0.52 ) eV.^[33]^ The spectrum of [Emin][DCA] was subtracted from the other spectra for further interpretation (see inset of Figure 3). An intensive adsorption band at about 300 nm is clearly visible in each difference spectrum, and a more or less pronounced shoulder towards longer wavelengths. Assuming that the difference spectra consist of two absorption bands, a sum of two Gaussian functions:

(1) $$f\left(\lambda \right)=\sum_{i=1}^{2}\frac{a_{i}}{\sqrt{2\pi }\sigma_{i}}\;e^{-\frac{{\left(\lambda -\lambda_{i}\right)}^{2}}{2\sigma_{i}^{2}}}$$

was used for quantitative description. The *a_i_* are scaling factors, *σ_i_* are the width parameters and *λ_i_* the peak positions. Curve fits employing Eq. (1) are in reasonable agreement with the experimental spectra, as shown in panels (a) to (c) of Figure 4. The resultant fit parameters are displayed in Figure 5. The maximum of $f_{1}\left(\lambda \right)$ has a mean value of $\lambda_{1}=\left(312\pm 2\right)$ nm and the mean value of the peak width is $\sigma_{1}=\left(32\pm 2\right)$ nm. Therefore, position and shape of $f_{1}\left(\lambda \right)$ are the same for all samples. For $f_{2}\left(\lambda \right)$ the situation is different: *λ*_2_ has a value of 354 nm for the clusters synthesized at 20 and 40 °C. For the clusters synthesized at 60 and 80 °C $\lambda_{2}\approx 400$ nm. Its peak width decreases from 55 nm to 14 nm with increasing synthesis temperature. The peak height at maximum increases from 0.8 to 1.2 for $f_{1}\left(\lambda \right)$ and decreases from 0.45 to 0.03 for $f_{2}\left(\lambda \right)$ . In other words, the contribution of $f_{2}\left(\lambda \right)$ to the spectrum decreases when the synthesis temperature decreases, which is the reason for the different visual appearances of the reaction products. UV‐Vis spectra of gold clusters are extensively described in the literature because clusters are considered key intermediates in studying transitions between molecular compounds and the bulk phase of gold. It has been shown that gold nanoparticles less than 2 nm in diameter behave molecule‐like instead of bulk‐like.^[34]^ For example, Fetzer et al.^[35]^ reported on phosphine‐protected Au_20_ metalloid gold clusters which display optical properties of molecular‐like behavior. The Au_20_ clusters display a strong peak at 320 nm which is assigned to intraband transitions (transitions from atomic states to super atomic states) and a peak at 419 nm assigned to interband transitions (transitions from superatomic states to superatomic states). Similarly, we suspect that the underlying optical transitions for $f_{1}\left(\lambda \right)$ may also result from intraband transitions and $f_{2}\left(\lambda \right)$ from interband transitions. We do not observe the typical surface plasmon resonance in the range of 520 to 540 nm which can be observed for gold nanoparticles of around 10 nm.^[36]^ Link and El‐Sayed investigated the size and temperature dependence of the plasmon absorption.^[37]^ Therein the smallest gold nanoparticles with a diameter of 8.9 nm display an absorption maximum at 517 nm. Recently, the surface plasmon red‐shift size dependence in the diameter range 3 to 10 nm has been reported by Sørensen et al.^[38]^ Therein, the particles with the smallest diameter of 3.13 nm only show a very weak and broad band at around 500 nm. In general, no surface plasmon resonance could be found in gold nanoparticles smaller than 2 nm in diameter.[^34^, ^39^] Thiol protected Au_25_ cluster crystal structures were related to their optical properties by Zhu et al.^[40]^ They stated that the quasi‐continuous electronic bands in larger gold nanoparticles (>5 nm) disappear and discrete levels evolve when the size of gold nanoparticles approaches the de Broglie wavelength of the conduction electrons (ca 1 nm diameter). Small gold nanoparticles (<3 nm) no longer display the bulk‐like electronic properties of 3–100 nm gold nanoparticles and no longer support the plasmon excitation characteristics. Similarly, ab initio calculations show that the threshold diameter of the metal core for localized surface plasmon is between 1.5 and 2.0 nm in monolayer protected gold clusters.^[41]^ Since no surface plasmon band is found in our UV‐Vis spectra, this is an indication of the presence of particles smaller than 3 nm.

We briefly discuss an alternative model for the interpretation of the UV‐Vis spectra. Therein, we assume that the signal obtained after subtracting the spectrum of pure [Emin][DCA] is mainly due to Au(III), with a peak around 315 nm and a shoulder between 350 and 450 nm (see e. g. Figure 1 of Peck et al.^[42]^). It should be noted that Fetzer et al.^[35]^ found a peak in the simulated spectra of Au_20_ clusters, but the peak was not present in their experimental data (Figure 3a of Ref. [35]). Further, it is well established in the literature (e. g. Ref. [34]) that even larger clusters Au_246_ with a diameter <2.2 nm display a very small plasmon peak. Therefore, the contribution of the gold clusters can be monotonic. Under this assumption, we tentatively model the UV‐vis difference spectra as the sum of a Gaussian and a monotonically decreasing function:

(2) $$f\left(\lambda \right)=\frac{a_{0}}{\sqrt{2\pi }\sigma_{0}}\;e^{-\frac{{\left(\lambda -\lambda_{0}\right)}^{2}}{2\sigma_{0}^{2}}}+a\lambda^{-n}$$

The *a*_0_ is the scaling factor of the Gaussian function, *σ*_0_ is the width parameter and *λ*_0_ the peak position. The *a* is the scaling factor and *n* is the exponent of the monotonically decreasing function. The resultant curves are shown in Figure S1. An overview of the model parameters of application of Eq. (1) and (2) are summarized in Table S1. It can be seen that the deviations of the second model from the data are much larger than for the first, i. e. the ratio of *χ*_2_ to *χ*_2_ is 6.64 at 20 °C, 6.42 at 40 °C, and 2.35 at 60 °C. Only at 80 °C, there is no preference for model 1 with $\chi_{2}^{2}/\chi_{1}^{2}=1$ . From the smaller *χ*‐values, we derive that *f*_2_ is more likely described by a Gaussian than by a monotonic function. For clarity, it should be noted that other interpretations of the UV‐Vis spectra are possible, especially since the UV‐Vis bands overlap considerably. Nevertheless, the presence of gold nanoparticles larger than 2 nm in diameter can be excluded because of the absence of a characteristic gold plasmon resonance band. Later in this work, more accurate sizing is performed using SAXS.

### Size of clusters

**Figure 6:**
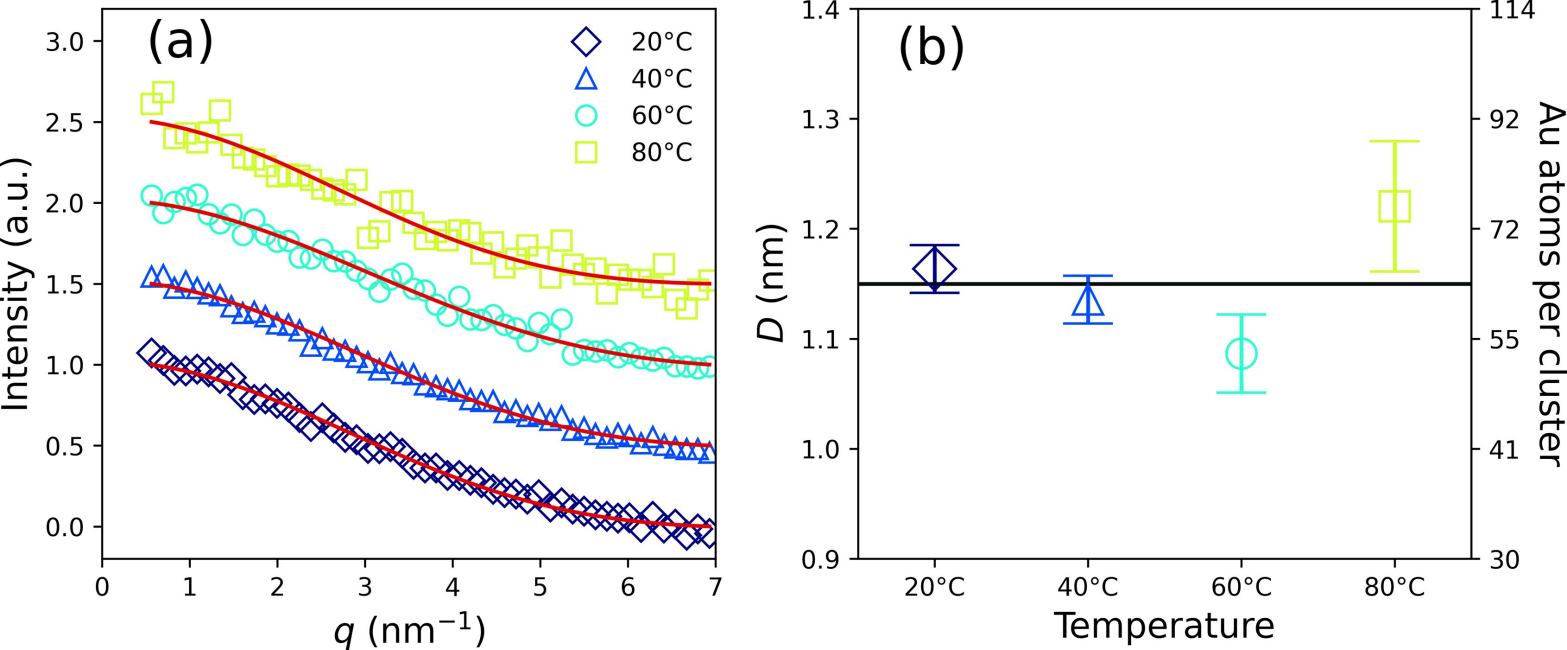
(a) SAXS data of clusters prepared at 20, 40, 60, and 80 °C for 24 h (symbols) and curve fits using the simple sphere model (red solid lines). (b) Diameters of the clusters corresponding to the curve fits in panel (a). Error bars represent one standard deviation. The horizontal line represents the mean diameter of the samples of $D_{\mathrm{mean}}=1.15$ nm and 62 gold atoms per cluster. The right y‐axis indicates the number of gold atoms per cluster.

The UV‐Vis measurements indicate but do not prove the presence of gold nanoclusters. SAXS measurements were carried out for this purpose, as this method allows for a reliable size determination of nanoparticles smaller than 10 nm directly in dispersion without error‐prone sample preparation.^[43]^ The resultant SAXS curves display the typical shape of very small particles, and we fitted the curves with a simple sphere model^[44]^ as can be seen in panel (a) of Figure 6 (symbols and red solid lines, respectively). The resultant diameters are (1.16±0.03 ) nm for a synthesis temperature of 20 °C, (1.13±0.02 ) nm for 40 °C, (1.09±0.04 ) nm for 60 °C and (1.22±0.06 ) nm for 80 °C. These diameters correspond to about 66±4 , 61±4 , 54±5 and 76±11 gold atoms per cluster if we assume a spherical shape of the clusters and a volume requirement of 0.0125 nm^3^ for a gold atom. It can thus be concluded that the clusters are in the size range suspected from the UV‐Vis measurements. The values for the diameters of the clusters are very similar within the limits of measurement accuracy. Furthermore, we cannot make any statement about the details of the structure of the clusters. At this point, we would like to comment on the apparently low data quality of the SAXS data. In this regard, it should be remembered that SAXS typically allows the estimation of particle diameters in the 1 nm to 100 nm size range (see international standard ISO 17867 : 2020). We are therefore at the lower end of the detection limit in the current work. A significantly better signal‐to‐noise ratio of the SAXS data is therefore hardly possible. We avoid an over‐interpretation of the SAXS data by using the simple sphere model, which has only two fit parameters: the forward scattering and the diameter.

**Figure 7:**
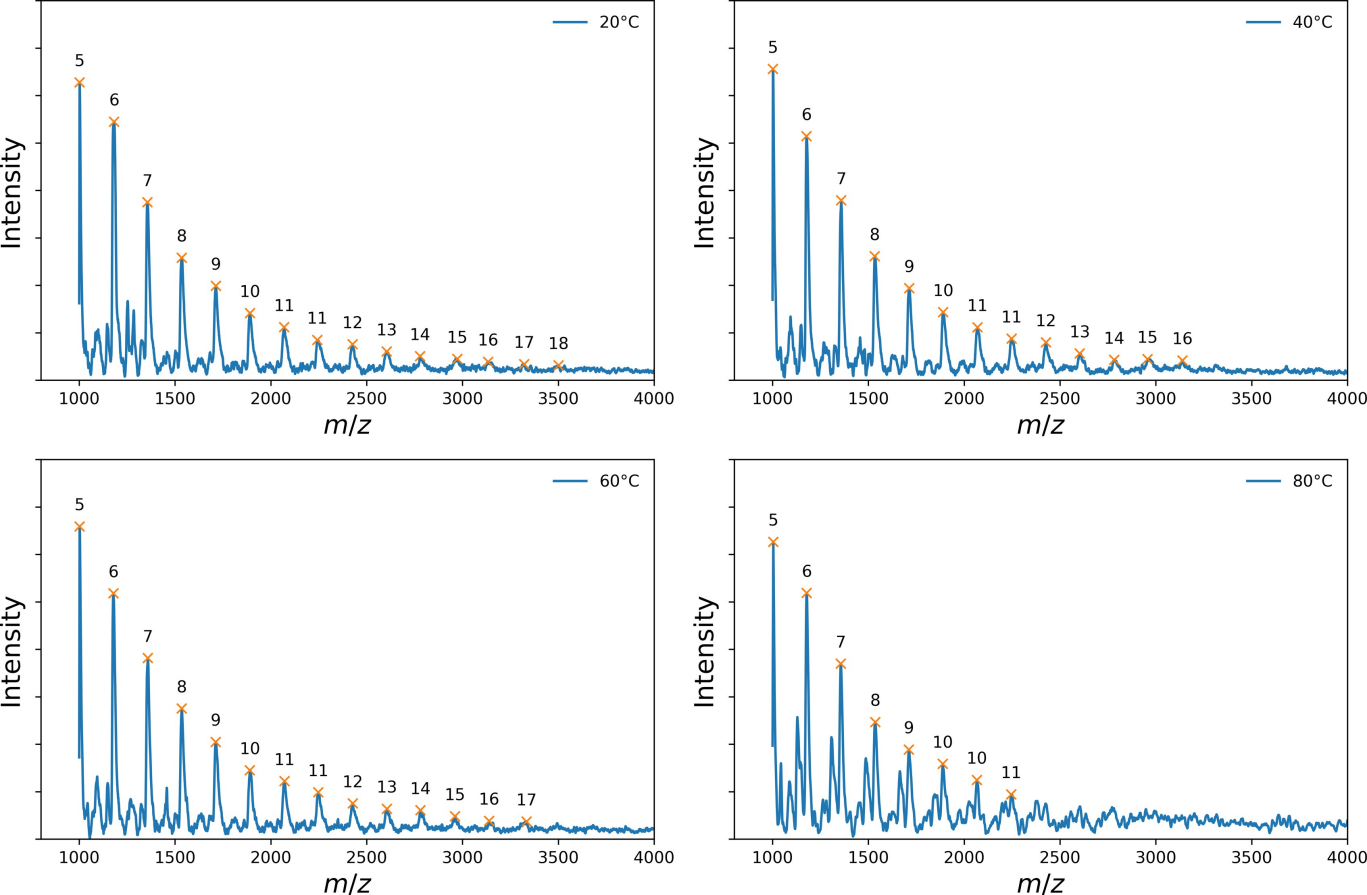
MALDI spectra from samples of the ILs containing gold clusters produced at temperatures of 20 °C, 40 °C, 60 °C and 80 °C. Markers at the peaks denote the number of gold atoms corresponding to the *m*/*z* ratio of the peaks for *z*=1.

The presence of gold clusters was further probed by matrix‐assisted laser desorption ionization time of flight (MALDI) mass spectrometry. Given clusters consisting of 54 to 76 gold atoms and neutral charge, peaks were expected in the range of 10638 *m*/*z* to 14972 *m*/*z*. However, numerous equidistant peaks are visible in the range of 1000 to 4000 *m*/*z* as shown in Figure 7 for all samples. Therein, we can assign the peaks to the masses of 5 to 18 gold atoms each. This supports the finding from the UV‐Vis and SAXS results that gold clusters were formed at all temperatures. The peak intensities decrease with increasing *m*/*z*‐ratio, which is an indication that the gold clusters are fragmenting. This could explain why the number of gold atoms of a cluster derived from SAXS is much larger than the number of gold atoms assigned to the peak in the MALDI spectra. Fragmentation of gold clusters in MALDI investigations does not seem to be unusual. For example, Pigliacelli et al. report on the fragmentation of 20 kDa superfluorinated clusters.^[45]^ Tsunoyama et al. provide a detailed MALDI mass analysis of 11 kDa gold clusters.^[46]^ They showed that gold cluster fragmentation can only be suppressed if elaborate and sample‐specific optimizations of the method are made.

We tried to obtain spectra with less fragmented clusters in a second series of MALDI experiments using DCBT as matrix and a higher acceleration voltage (see Experimental). The resultant spectra display broad distributions with maxima at m/z‐values around 2500 and wide tails towards 20000 (see Figure S2). The resolution in the spectra is lower than in the first series of measurements so that the mass differences of individual gold atoms can no longer be detected, but the broad peaks at relatively large *m*/*z*‐values suggest that the spectra result from less fragmented clusters. For a comparison with the diameter resulting from the SAXS data, it should be taken into account that the MALDI spectra are number‐weighted. In contrast, the SAXS data are intensity weighted. To quantify the MALDI spectra, we fitted them with a lognormal distribution (see red solid lines in Figure S2). Fit parameters are median ${\left(m/z\right)}_{median}$ and width parameter *σ*. The ${\left(m/z\right)}_{median}$ ‐values are between 4051 and 5387, the *σ* between 0.64 and 0.76. The mean values, calculated by ${\left(m/z\right)}_{mean}=e^{\frac{\sigma^{2}}{2}}\;{\left(m/z\right)}_{median}$ , range between 5419 and 6631 and correspond to the mass of 28 and 34 gold atoms. These values are closer to the numbers determined with SAXS. Continuing to look at the volume‐ and intensity‐weighted mean (see Table S2) suggests that there is no discrepancy between the MALDI and SAXS results.

For the present study, it is sufficient to establish that gold clusters are indeed present in all samples.

### Kinetics of cluster formation

**Figure 8:**
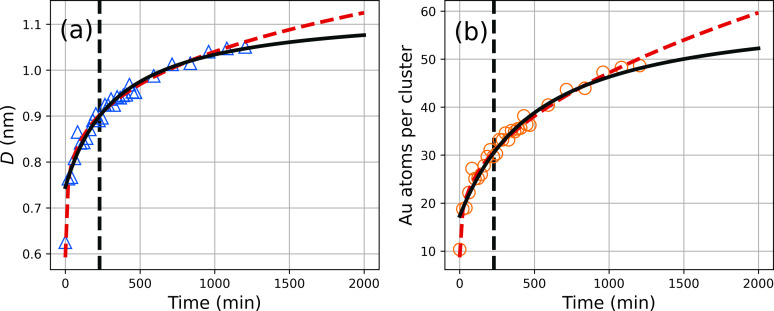
(a) Diameter of clusters as a function of the reaction time and curve fits according to Eqs. (3) and (4) (red dashed and black solid line, respectively). (b) Number of gold atoms per cluster as a function of time (symbols) as derived from the cluster diameter shown in panel (a) assuming a spherical shape of the clusters and a volume requirement of *v*_Au_=1.25×10^−2^ nm^3^ per gold atom. The vertical black dashed lines indicate *t*_0_ of the oriented attachment. The reaction temperature was 40 °C.

The results so far indicate that the clusters final sizes do not significantly depend on the synthesis temperature. Therefore, we select a representative synthesis temperature of 40 °C to study cluster formation with SAXS as a function of time. The first SAXS measurement was completed ca. 10 minutes after preparing the reaction mixture (time point t=0 ). The evaluation of the measured SAXS data provided the clusters diameter and number of gold atoms per cluster (see Figure 8a and 8b, respectively). It can be seen that the diameter is D=0.62 nm at t=0 , which corresponds to 9–10 gold atoms. After an initial strong increase in diameter, the growth slows at longer times, as was expected, since we know that the size of the final clusters is stable over time. The number of gold atoms per cluster of $n_{\mathrm{Au}}=\pi D^{3}/\left(6v_{\mathrm{Au}}\right)$ was estimated by assuming a spherical shape of the clusters and a volume requirement of $v_{\mathrm{Au}}=1.25\times 10^{-2}$ nm^3^ per gold atom. We model the cluster diameter *D*(*t*) in the cluster growth phase in terms of the model derived by Lifshitz, Slyozov^[47]^ and Wagner,^[48]^ also known as the LSW model:

(3) $$D\left(t\right)=D_{0}+k_{\mathrm{LSW}}\;t^{1/n}$$

where *D*_0_ is the diameter of the particle at t=0 , *k*_LSW_ is a temperature‐dependent material constant and *n* is an integer that can take the values 2, 3 or 4 depending on how growth is controlled.^[49]^ In the present study, the parameters *D*_0_ and *k*_LSW_ were optimized freely, while the value of *n* was iterated over the integer values 2, 3, and 4 at curve fitting. The lowest reduced sum of squared residuals of $\chi_{r}=0.47$ was obtained for $D_{0}=\left(0.59\pm 0.01\right)$ nm, $k_{\mathrm{LSW}}=\left(8.0\pm 0.1\right)\times 10^{-2}$ nm min^−1/*n*^ and n=4 . The latter value indicates a cluster growth mechanism of dissolution of smaller clusters at the cluster‐solution interface. This indicates an Ostwald ripening cluster growth, which was reported for the growth of nanocrystalline lead sulfide by Brazeau and Jones.^[49]^ If the LSW model is appropriate, then the number density *n* of the clusters should decrease with time and the total mass concentration of clusters *c* should be constant. This can be verified as the following properties apply: $n\propto I_{0}/V^{2}$ and $c\propto I_{0}/V$ , where *I*_0_ is the scattering intensity at small values of the scattering vector and *V* is the cluster volume. The relative temporal changes of *N* and *c* were determined accordingly and are shown in Figure S3. Indeed, it can be seen that *n* decreases with time, and *c* is largely constant. Both findings support the assumption that the LSW model applies.

The LSW model according to Eq. (3) is satisfactory for interpreting the cluster growth over a wide period of up to about 800 minutes (see red dashed lines in Figure 8a and 8b). But the LSW model predicts infinite growth. Therefore, we need a second mechanism for long reaction times that limits cluster growth. We tentatively assume that clusters combine via an oriented attachment without the dissolution of either cluster to form a colloidally stable larger cluster. Huang et al.^[50]^ provided an expression for this coarsening process as:

(4) $$D\left(t\right)=D_{H}\frac{\sqrt[{3}]{2}\;k_{H}\left(t-t_{0}\right)+1}{k_{H}\left(t-t_{0}\right)+1}$$

where *D*_H_ is the diameter of the initial cluster at time *t*_0_ and *k*_H_ is the rate constant for the oriented attachment. Application of Eq. (4) for fitting the data provides $D_{H}=\left(0.90\pm 0.01\right)$ nm, $k_{H}=\left(1.74\pm 0.29\right)\times 10^{-3}$ s^−1^ and $t_{0}=\left(228\pm 40\right)$ min (see black solid line in Figure 8a). The final cluster size is $D\left(\infty \right)=2^{1/3}D_{H}=\left(1.13\pm 0.02\right)$ nm consisting of $\left(61\pm 3\right)$ gold atoms. When considering Eq. (4) the growth is 94 % completed after 24 h which is close to its theoretical maximum and is in agreement with our observation of colloidal stable cluster dispersion. An overview of our proposal of how the cluster formation proceeds via nucleation, Ostwald ripening, and oriented attachment can be found in Table 1. Of course, other scenarios of clustering are also conceivable, for which we refer to the work of Wang et al., who reviewed aggregative growth as a nonclassical nucleation and growth process.^[51]^ Further, Keller et al.^[52]^ investigated the atomic mechanisms of gold nanoparticle growth in ILs, studied by in situ scanning transmission electron microscopy. They confirmed Ostwald ripening and oriented particle coalescence according to the mechanisms suggested by theory. Moreover, however, they also identified unexpected growth phenomena and more intricate coalescence events which show competing mechanisms. They concluded that the diversity of the observed growth processes thus illustrates that growth reactions in liquids, on the atomic scale, are much more complex than predicted by theory. Koerner et al. employed the LSW model for the interpretation of gold nanoparticle growth based on in situ UV‐Vis, SAXS, and TEM investigations.^[53]^ The particle growth in their study was stopped by thiol capping agents of different alkyl chain lengths. The gold nanoparticles in their study display radii in the range of 4 to 6 nm, which corresponds to 2000–9000 gold atoms per nanoparticle. This large difference in the number of gold atoms per nanoparticle in the study of Koerner et al.^[53]^ and gold atoms per cluster in our study is probably the reason why we were not able to produce TEM images of the clusters.

**Table 1:** Overview of parameters of cluster formation derived from SAXS and UV‐Vis data, respectively, at a reaction temperature of 40 °C.

Mechanism	Ostwald ripening	Oriented attachment
method	SAXS	
model	$D\left(t\right)=D_{0}+k_{\mathrm{LSW}}\;t^{1/n}$	$D\left(t\right)=D_{H}\frac{\sqrt[{3}]{2}\;k_{H}\left(t-t_{0}\right)+1}{k_{H}\left(t-t_{0}\right)+1}$
parameters	$D_{0}=\left(0.59\pm 0.01\right)$ nm	$D_{H}=\left(0.90\pm 0.01\right)$ nm
		$D\left(\infty \right)=\left(1.13\pm 0.02\right)$ nm
	$k_{\mathrm{LSW}}=\left(8.0\pm 0.1\right)\times 10^{-2}$ nm min^−1/*n*^	$k_{H}=\left(1.74\pm 0.29\right)\times 10^{-3}\;\min^{-1}$
	n=4	$t_{0}=\left(228\pm 40\right)$ min
		
method	UV‐Vis spectroscopy	
model	$a_{2}\left(t\right)=a_{0}+k_{\mathrm{LSW},\mathrm{UV}}\;t^{1/n}$	$a_{2}\left(t\right)=a_{H,\mathrm{UV}}\frac{\sqrt[{3}]{2}\;k_{H,\mathrm{UV}}\left(t-t_{0,\mathrm{UV}}\right)+1}{k_{H,\mathrm{UV}}\left(t-t_{0,\mathrm{UV}}\right)+1}$
parameters	$a_{0}=0.06\pm 0.02$	$a_{H,\mathrm{UV}}=0.40\pm 0.02$
	$k_{\mathrm{LSW},\mathrm{UV}}=\left(7.9\pm 0.5\right)\times 10^{-2}$ min^−1/*n*^	$k_{H,\mathrm{UV}}=\left(2.1\pm 0.4\right)\times 10^{-3}$ min^−1^
	n=4	$t_{0,\mathrm{UV}}=\left(332\pm 57\right)$ min
		
time	t<500-800 min	200–350 min <t<24 h
gold atoms	10≤i≤31	i=62±3

**Figure 9:**
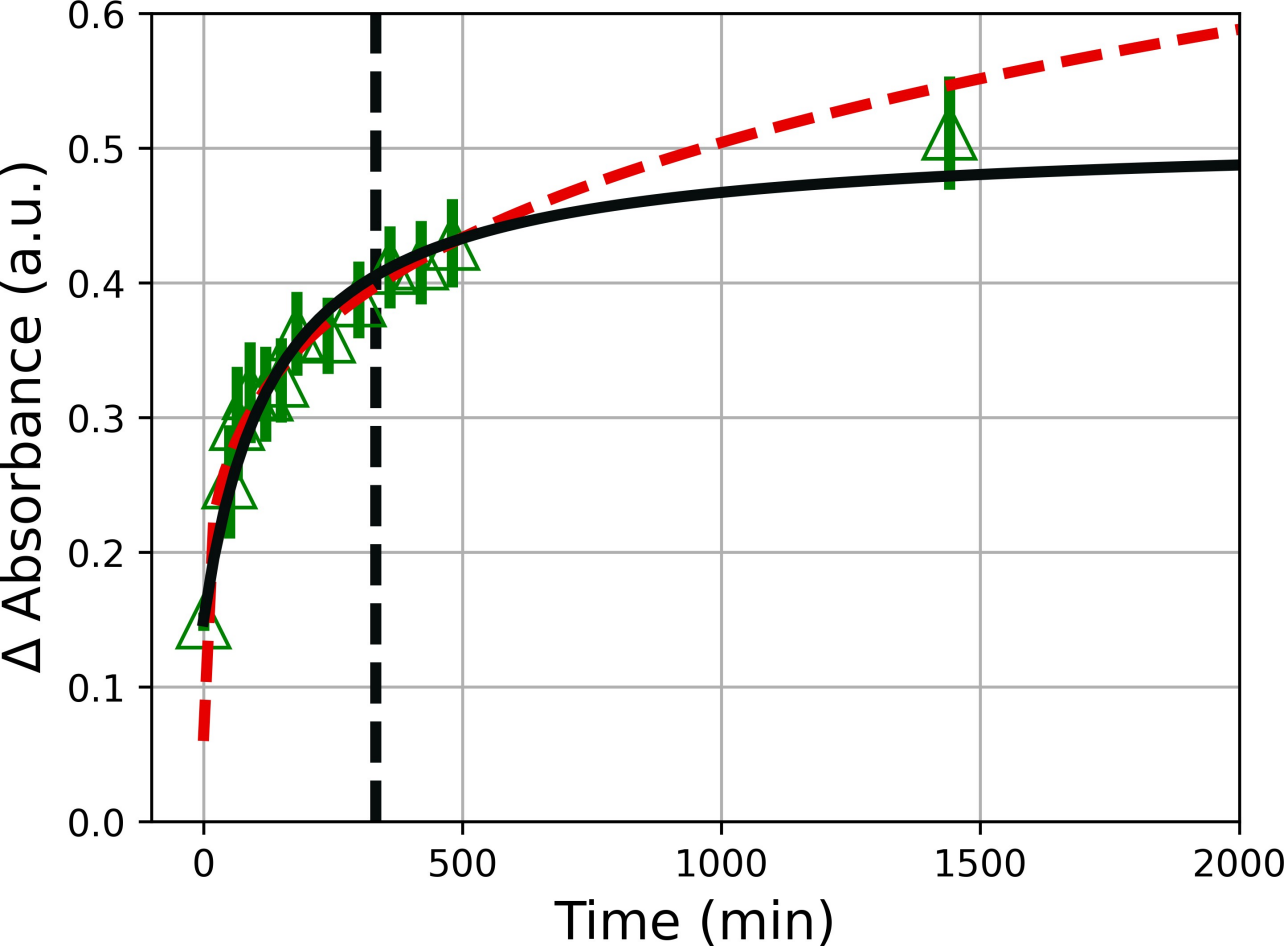
Change of the absorbance of band $f_{2}\left(\lambda \right)$ of the of clusters as a function of the reaction time and curve fits according to Eqs. (3) and (4) (red dashed and black solid line, respectively). The vertical black dashed line indicates *t*_0,UV_ of the oriented attachment. The reaction temperature was 40 °C.

To provide further evidence for the proposed mechanism for cluster formation, time‐dependent UV‐Vis measurements were performed at 40 °C. The resultant UV‐Vis absorption spectra of [Emim][DCA] and gold nanoclusters in [Emim][DCA] as a function of time are shown for comparison in Figure S4. The inset in Figure S4 displays the difference spectra in which the spectrum of [Emim][DCA] has been subtracted. The spectrum at a time of 0 minutes was measured after the complete dissolution of the gold salt (10 minutes after mixing of HAuCl_4_ ⋅ 3 H_2_O with [Emim][DCA]). The UV‐Vis difference spectra were fitted with Eq. (1) (not shown), providing the curve fit parameters of $f_{1}\left(\lambda \right)$ and $f_{2}\left(\lambda \right)$ as a function of time (see Figure S5). It can be seen that the wavelength at the peak maximum and the peak width of $f_{1}\left(\lambda \right)$ are constant. By contrast, the peak maximum of $f_{2}\left(\lambda \right)$ passes through a minimum and the peak width passes through a maximum at a reaction time of about 200 min. The absorption of $f_{1}\left(\lambda \right)$ decreases initially and approaches a constant value at long reaction times. In contrast, the absorption of $f_{2}\left(\lambda \right)$ initially increases rapidly and also approaches a constant value. It seems reasonable to assume that $f_{2}\left(\lambda \right)$ reflects the growth of the clusters. The shape of the time course of the absorption of $f_{2}\left(\lambda \right)$ is similar to that of the cluster diameters. Therefore, we adapted Eqs. (3) and (4) for curve fitting of the absorbance of $f_{2}\left(\lambda \right)$ as a function of time (see dashed red and solid black line in Figure 9). For the LSW model – here $a_{2}\left(t\right)=a_{0}+k_{\mathrm{LSW},\mathrm{UV}}\;t^{1/n}$ – we found $a_{0}=0.06\pm 0.02$ , n=4 and $k_{\mathrm{LSW},\mathrm{UV}}=\left(7.9\pm 0.5\right)\times 10^{-2}$ min^1/*n*^. For the oriented attachment – $a_{2}\left(t\right)=a_{H,\mathrm{UV}}\frac{\sqrt[{3}]{2}\;k_{H,\mathrm{UV}}\left(t-t_{0,\mathrm{UV}}\right)+1}{k_{H,\mathrm{UV}}\left(t-t_{0,\mathrm{UV}}\right)+1}$ – we found $a_{H,\mathrm{UV}}=0.40\pm 0.02$ , $t_{0,\mathrm{UV}}=\left(332\pm 57\right)$ min and $k_{H,\mathrm{UV}}=\left(2.1\pm 0.4\right)\times 10^{-3}$ min^−1^. Comparison of the values of $k_{\mathrm{LSW},\mathrm{UV}}$ with *k*_LSW_ shows that the rate constants are very similar. The same applies for *t*_0_ and $t_{0,\mathrm{UV}}$ . This indicates that the growth of the clusters and the UV absorption band $f_{2}\left(\lambda \right)$ are directly related.

## Conclusions

Colloidally long‐term stable dispersions of gold clusters with a diameter of ca. 1 nm were prepared in an IL. Surprisingly, no addition of a stabilizer in the form of a surfactant is necessary to stabilize the clusters. Apparently, the IL takes on the dual function of reducing agent and stabilizer. Fine‐tuning of the visible appearance is possible from red to light orange by varying the synthesis temperature between 20 °C and 80 °C. The kinetics of cluster formation was investigated at a temperature of 40 °C and interpreted with a three‐step model. The cluster formation starts with a fast reduction of gold ions and the formation of cluster nuclei (consisting of <9 gold atoms) already during the mixing of the reaction mixture. The second step in cluster formation is an Ostwald ripening process that follows the kinetics of the LSW‐Model up to ca 500–800 min. Here, the number of gold atoms is 10 to 31 in the growing cluster. In the third and final step, two growing clusters combine into the final and stable clusters consisting of 62±3 gold atoms via an oriented attachment. A summary is provided in Table 1. The results of the present work could serve as a starting point for the systematic investigation of the properties of metal clusters dispersed in a liquid phase.

## Experimental Section

### Materials

Tetrachloroauric (III) acid trihydrate (HAuCl_4_ ⋅ 3 H_2_O) was obtained from Sigma Aldrich (≥99.9 %). 1‐Ethyl‐3‐methylimidazolium dicyanamide ([Emim][DCA], >98 %) was purchased from Iolitec Ionic Liquids Technologies GmbH. All chemicals were used as received without further purification.

### Synthesis of gold nanoclusters

An amount of 16 mg of tetrachloroauric (III) acid trihydrate (HAuCl_4_ ⋅ 3 H_2_O) was added to 1 L of the IL 1‐ethyl‐3‐methylimidazolium dicyanamide ([Emim][DCA]) in a screw‐top bottle (*c*_Au_=8 mg mL^−1^). The gold salt was dissolved via stirring at room temperature. After the complete dissolution of the salt, the bottle was stored for up to 24 hours at the respective temperature. The synthesis was carried out at 20, 40, 60 and 80 °C. For the temperatures of 40–80 °C, an appropriately pre‐tempered drying oven was used. To obtain the UV‐Vis measurement series, small amounts of samples were taken and measured at the respective time during the synthesis. These aliquots were then discarded. For the SAXS measurement series, the samples were inserted into the instrument after dissolving the salt and the sample holder was heated to the desired temperature so that the reaction could be observed directly.

### Small‐angle X‐ray scattering

Small‐angle X‐ray scattering (SAXS) measurements were performed in a polycarbonate flow‐through capillary at 21 °C with a SAXSess camera (Anton Paar, Austria). This camera was attached to a laboratory X‐ray generator (PW3830, PANalytical) and operated with a fine focus glass X‐ray tube at a voltage of 40 kV and a current of 40 (Cu‐$K_{\alpha }$ , *λ*=0.1542 nm). A focusing multilayer optics and a block collimator provided a monochromatic primary beam with low background noise. The samples were measured in a glass capillary with 1 mm in diameter. The pure IL was measured in the same capillary. The scattering from the pure IL was subtracted as background from the measurements.

### UV‐Vis measurements

UV‐Vis measurements were performed using a StellarNet Inc. BLACK‐Comet C‐50 Spectrometer with an SL5 Deuterium+Halogen Light Source. A Hellma demountable quartz cuvette (QS) with a 0.10 mm light path was used. The measurements were integrated over a period of 50 s and averaged for 5 cycles.

### MALDI mass spectrometry

An AutoflexMaX (Bruker Daltonik GmbH, Bremen Germany) was used. For the first measurement series, the AU samples were used as received and dropped (1 μL) on a stainless steel target. After drying, the sample holder was inserted and samples were irradiated with a Ny‐YAG Smartbeam laser working at 355 nm and 1000 Hz. Typically, 1000 laser shots from 4 different places of the spot were accumulated to a spectrum. Calibration was done using peptide standards (Bruker). FlexControl and FlexAnalysis (Bruker) were used for recording and calculating raw data.

Conditions for the second measurement series were as follows: The instrument was an AutoflexMax (Bruker) operating at the same conditions (laser etc.) as in the first series but with a frequency of 2000 Hz. The acceleration voltage was 20 kV, a number of 2000 shots were accumulated for a spectrum, and DCTB (trans‐2‐[3‐(4‐tert‐butylphenyl)‐2‐methyl‐2‐propenyliden]‐malononitril) (Fluka) was used as matrix.

## Supporting Information Summary

The Supporting Information includes UV‐Vis absorption difference spectra and curve fits. Overview of parameters of model the UV‐Vis data of clusters. Spectra of the second series of MALDI experiments. MALDI fit parameters. Normalized number density *n* and weight concentration *c* of clusters as a function of the reaction time. UV‐Vis absorption spectra of [Emim][DCA] and gold nanoclusters in [Emim][DCA] as a function of time. Parameter of absorption band 1 and 2 found in the UV‐Vis spectra in the course of 24 hours.

## Conflict of interest

The authors declare no conflict of interest to declare.

1

## Supporting information

As a service to our authors and readers, this journal provides supporting information supplied by the authors. Such materials are peer reviewed and may be re‐organized for online delivery, but are not copy‐edited or typeset. Technical support issues arising from supporting information (other than missing files) should be addressed to the authors.

Supporting Information

## Data Availability

The data that support the findings of this study are available from the corresponding author upon reasonable request.
