# Supplementary material for: Synthesis and Characterization of Ultra‐Small Gold Nanoparticles in the Ionic Liquid 1‐Ethyl‐3‐methylimidazolium Dicyanamide, [Emim][DCA]
Source: ChemistryOpen. 2023 Aug 31;13(2):e202300106. doi: 10.1002/open.202300106 (PMC10853075; doi:10.1002/open.202300106)
Supplement: Supplementary file 1 — Supporting Information [file OPEN-13-e202300106-s001.pdf]

ChemistryOpen

Supporting Information

Synthesis and Characterization of Ultra-Small Gold Nanoparticles in the Ionic Liquid 1-Ethyl-3-methylimidazolium Dicyanamide, [Emim][DCA]

Jana Hildebrandt, Andreas Taubert,* and Andreas F. Thünemann*

UV-Vis: Alternative model

MALDI

The spectra of the second series of MALDI experiments as shown in Figure S2 were fitted with a lognormal distribution

$$I(m/z) = \frac{k}{(m/z)\sigma\sqrt{2\pi}} \exp - \left(\frac{\log\left[(m/z)\right] - \log\left[(m/z)_{median}\right]}{2\sigma^2} \right) \tag{S1}$$

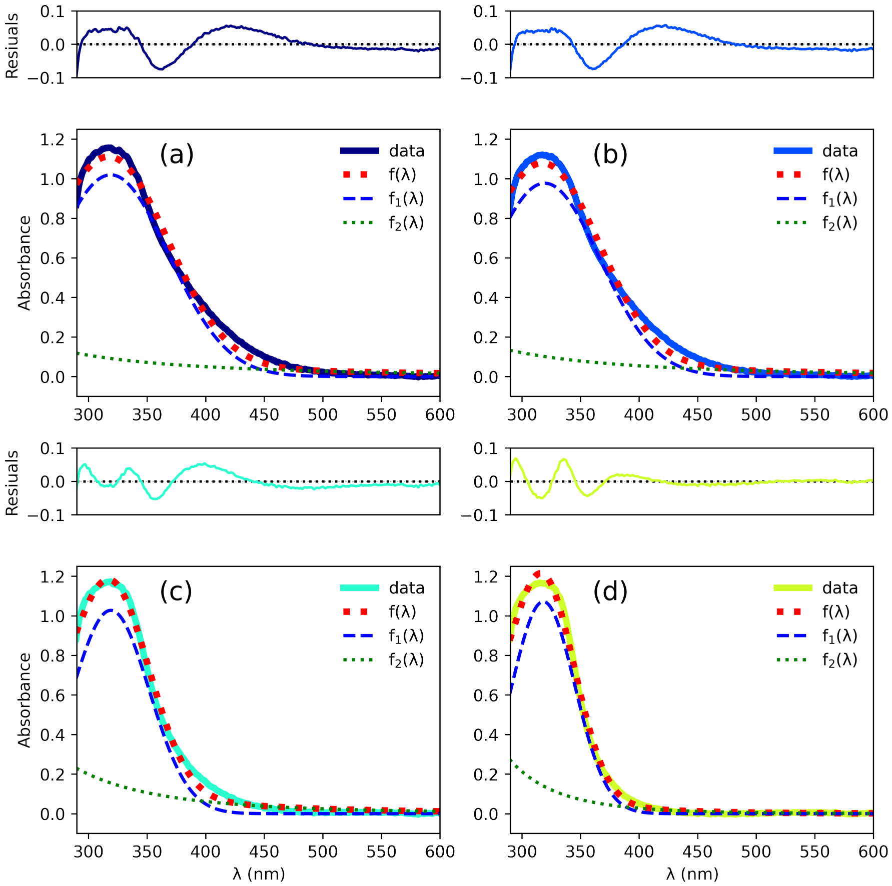

Figure S1: UV-Vis absorption difference spectra (solid lines) and curve fits $f(\lambda)$ using eq. (2) (red dotted lines) for clusters synthesized at 20 °C (a), 40 °C (b), 60 °C (c) and 80 °C (d). Contributions $f_1(\lambda)$ and $f_2(\lambda)$ are given (blue dashed and green dotted lines, respectively) The differences between the data and the model curves are shown above each panel.

Table S1: Overview of parameters of model the UV-Vis data of clusters synthesized at reaction temperatures of 20 °C, 40 °C, 60 °C, and 80 °C Parameters resulting from application of eq. (1) are a_1, λ_1, σ_1, a_2, λ_2 and σ_2. Parameters from eq. (2) are a_0, λ_0, σ_0, a and n. Indicators for comparison of the curve fits are χ_1^2, χ_2^2, and the ratios χ_2^2/χ_1^2.

T ($^\circ C$)	a_1	λ_1 (nm)	σ_1 (nm)	a_2	λ_2 (nm)	σ_2 (nm)	a_0	λ_0 (nm)	σ_0 (nm)	a	n	χ_1^2	χ_2^2	$\frac{\chi_2^2}{\chi_1^2}$
20	0.83	312.0	32.4	0.45	354.0	55.1	1.02	319.0	49.4	4e+05	2.7	0.16	1.05	6.64
40	0.81	312.3	32.0	0.42	353.7	54.5	0.98	319.4	47.3	8e+05	2.8	0.15	0.99	6.42
60	1.20	316.2	33.5	0.10	401.2	26.5	1.03	319.1	32.8	3e+09	4.1	0.21	0.49	2.35
80	1.22	314.7	29.7	0.03	398.7	13.9	1.07	318.3	26.8	8e+16	7.1	0.33	0.33	1.00

with a scaling factor k, a median value $(m/z)_{median}$ and a width parameter σ. Curve fits utilizing eq. (S1) are displayed in Figure S2 (red solid lines). The MALDI spectra represent a number-weighted distribution (even if this does not represent the "real" number-weighted distribution of the clusters in the sample because of the MALDI measurement process). It should be noted that in the case of broad distributions - as found here - it is important for comparison that often not number-weighted but volume- or intensity-weighted distributions are obtained with other methods. SAXS data, for example, are intensity-weighted. The differently weighted distributions can in principle be converted into each other. However, this often results in large uncertainties. For simplicity, we calculate the mean of the distribution with the respective weighting. The $(m/z)_{median}$ and σ-values as obtained from the curve fits define the shape of the distribution and were used to estimate the mean values of the number-, volume- and intensity-weighted distribution. The number-weighted mean values of the lognormal distribution were calculated as the volume-weighted (weighted with $(m/z)^3$) mean as $e^{\frac{7\sigma^2}{2}}\,(m/z)_{median}$ and the intensity-weighed (weighted with $(m/z)^6$) as $e^{\frac{13\sigma^2}{2}}\,(m/z)_{median}$. The results are summarized in Table S3.

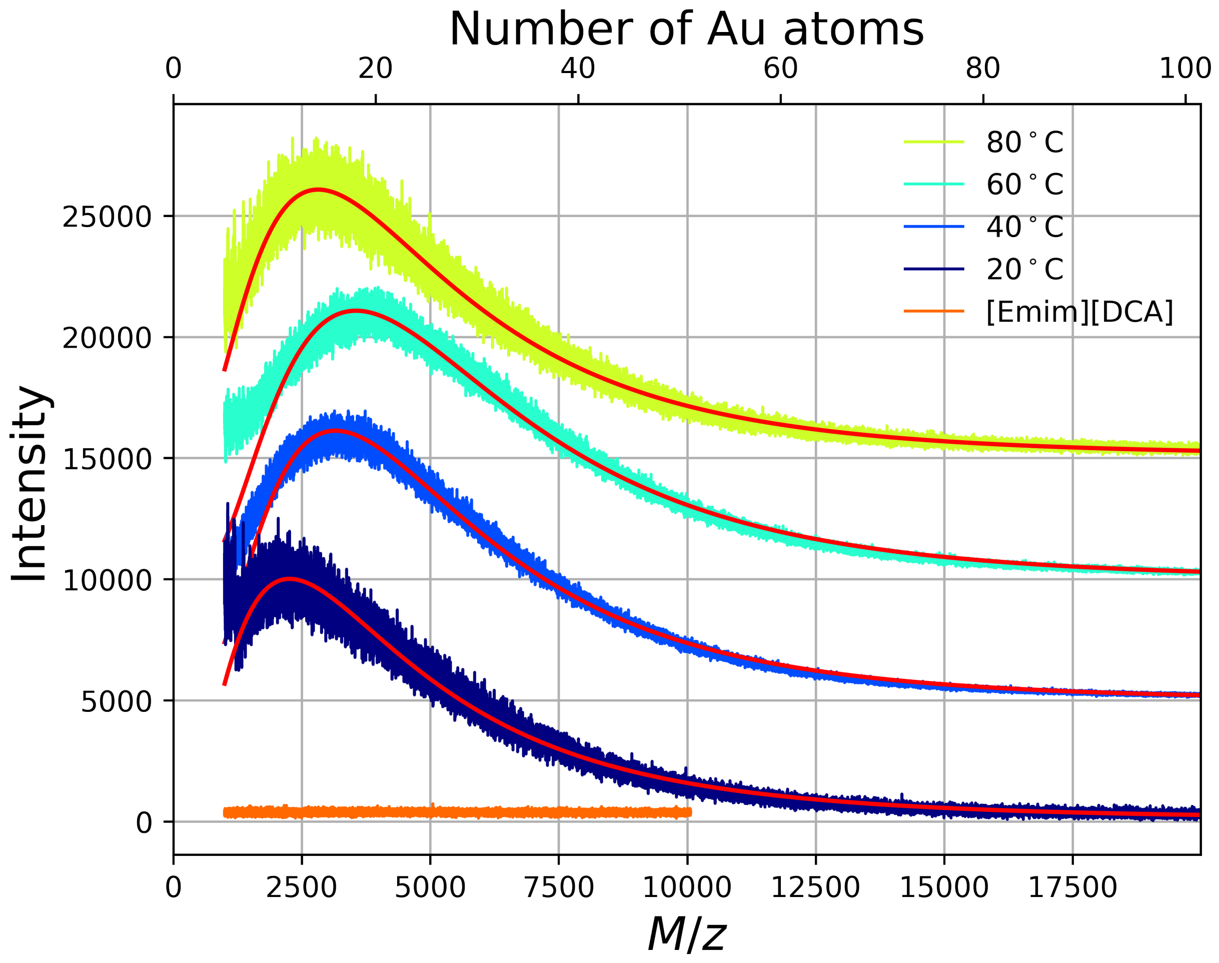

Figure S2: MALDI spectra from samples of the ILs containing gold clusters produced at temperatures of 20 °C, 40 °C, 60 °C and 80 °C. The spectrum of [Emin][DCA] is given for comparison. Curve fits utilizing eq. (S1) are given (red solid lines). Curves are vertically shifted for visualization.

Table S2: MALDI fit parameters $(m/z)_{median}$ and σ of the second measurement series, utilizing a lognormal distribution for interpretation of the intensity as a function of the m/z-values. Given are also the mean (m/z)-values of the number-, volume- and intensity-weighted distributions and the corresponding number of gold atoms per cluster n_{Au}.

T (°C)	$(m/z)_{median}$	σ	number-weighted $(m/z)_{mean}$ (n_{Au})	volume-weighted $(m/z)_{mean}$ (n_{Au})	intensity-weighted $(m/z)_{mean}$ (n_{Au})
20	4051	0.76	5419 (28)	31051 (158)	177921 (903)
40	4847	0.66	6008 (31)	21793 (111)	79052 (401)
60	5387	0.64	6631 (34)	23061 (117)	80197 (407)
80	4546	0.69	5771 (29)	24168 (123)	101211 (514)

SAXS: Kinetics of cluster formation

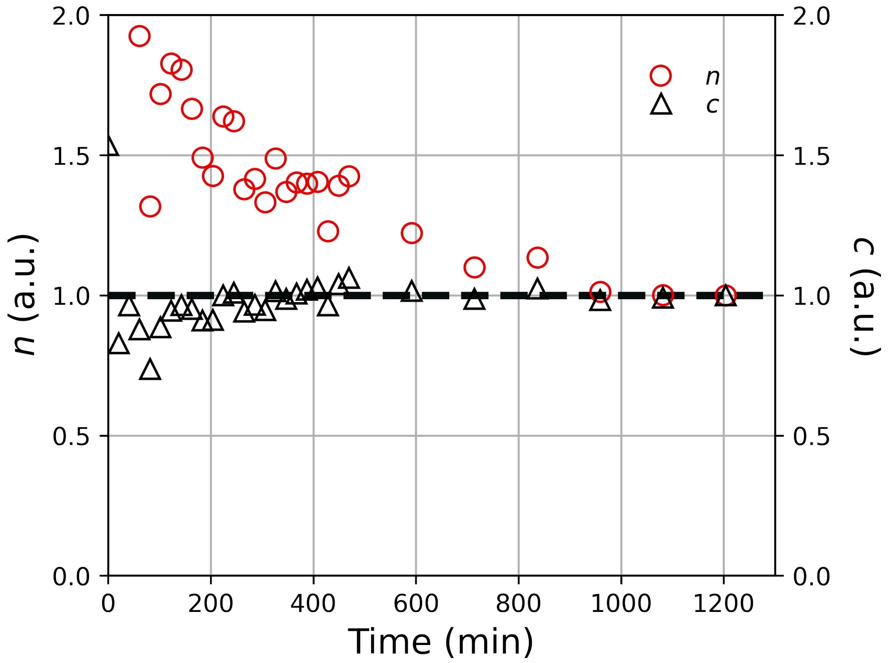

Figure S3: Normalized number density n and weight concentration c of clusters as a function of the reaction time at 40 °C (red circles and black triangles, respectively). The values are normalized to the last data point.

UV-Vis: Kinetics of cluster formation

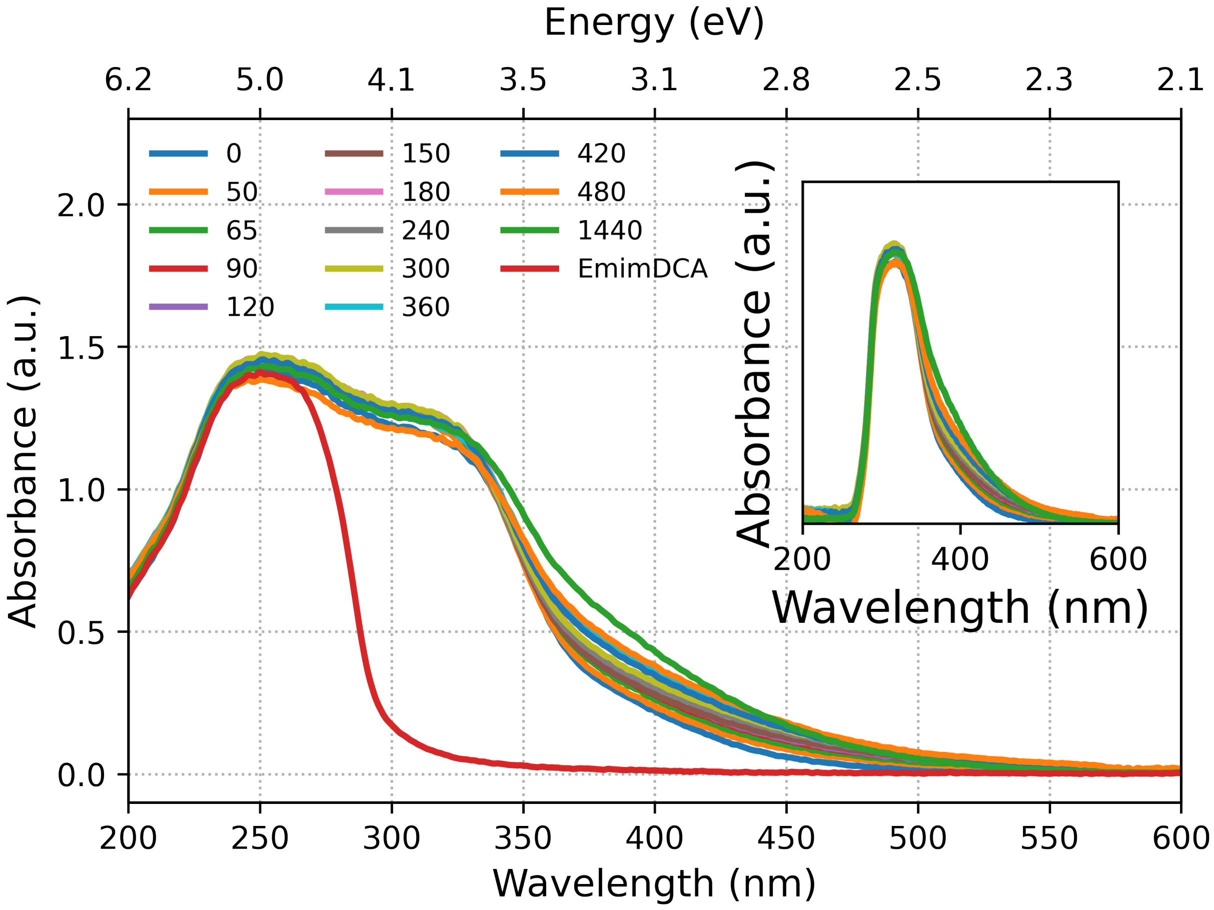

Figure S4: UV-Vis absorption spectra of [Emim][DCA] and gold nanoclusters in [Emim][DCA] as a function of time. The temperature was 40 °C. Inset: Difference spectra, i.e. spectra in which the spectrum of [Emim][DCA] has been subtracted. The spectrum at a time of 0 minutes was measured after the complete dissolution of the gold salt (10 minutes after mixing of $HAuCl_4 \cdot 3\ H_2O$ with [Emim][DCA].

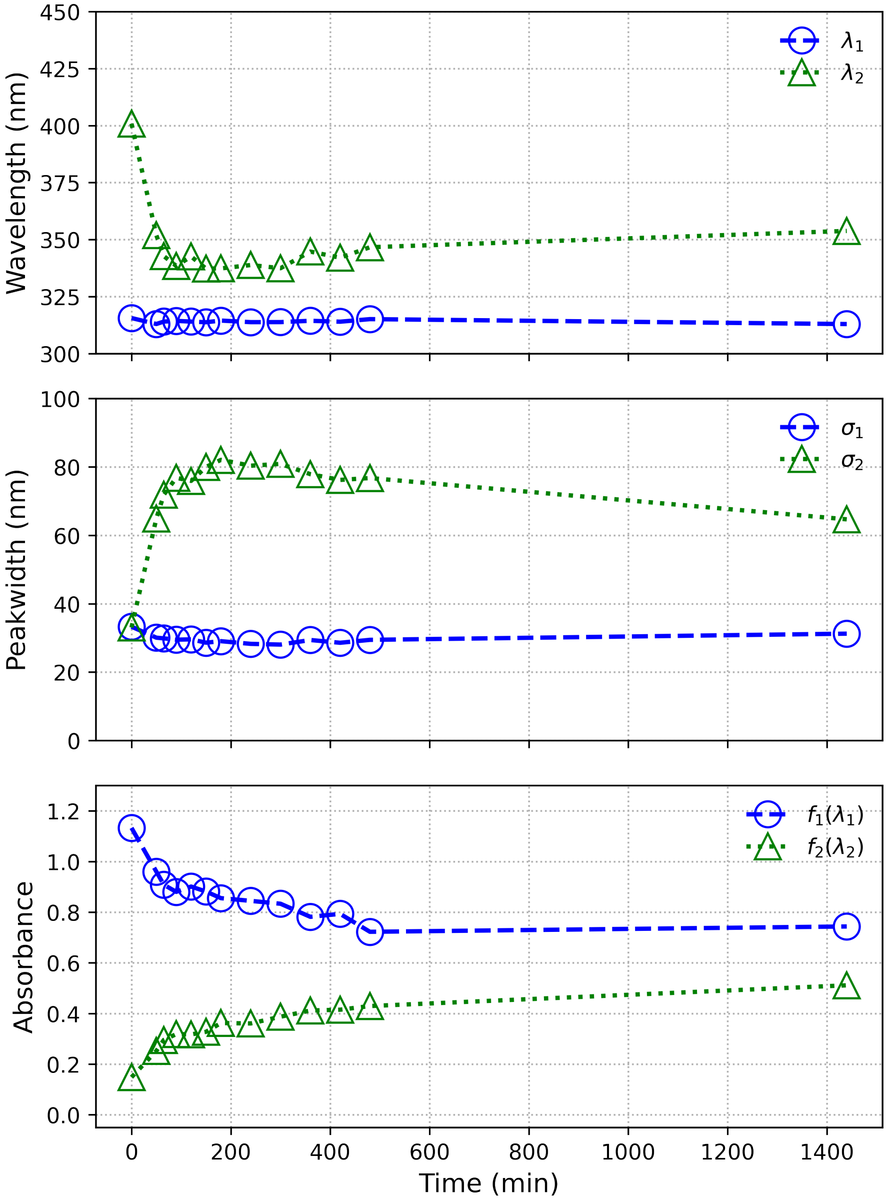

Figure S5: Kinetics of cluster formation at 40 °C. Parameter of absorption band 1 and 2 found in the UV-Vis spectra in the course of 24 hours.
